# Involvement of Tim-3 in Maternal-fetal Tolerance: A Review of Current Understanding

**DOI:** 10.7150/ijbs.106115

**Published:** 2025-01-01

**Authors:** Xinhang Meng, Yujie Luo, Liyuan Cui, Songcun Wang

**Affiliations:** Laboratory for Reproductive Immunology, Hospital of Obstetrics and Gynecology, Fudan University Shanghai Medical College, Shanghai 200011, China.

**Keywords:** Tim-3, Maternal-fetal Tolerance, Pregnancy

## Abstract

As the first T cell immunoglobulin mucin (Tim) family member to be identified, Tim-3 is a powerful immune checkpoint that functions in immunoregulation and induction of tolerance. Conventionally, Tim-3 is considered to play a role in adaptive immunity, especially in helper T cell-mediated immune responses. As researches progress, Tim-3 has been detected in a wider range of cell types, modulating cell function through ligand-receptor interactions and other pathways. Strikingly, Tim-3 plays a pivotal role in maternal-fetal tolerance by regulating immune cell functions and orchestrating the maternal-fetal cross-talk. In this review, we elaborate on the involvement of Tim-3 in immunology, with a focus on its participation in maternal-fetal tolerance to provide new insights into immunoregulation during pregnancy. Our work will be helpful in further understanding the pathogenesis of pregnancy-related diseases and will inspire new strategies for their diagnosis and treatment.

## Introduction

T cell immunoglobulin mucin-3 (Tim-3), also known as the hepatitis A virus cellular receptor 2 (HAVCR2) protein or cluster of differentiation (CD)366, is a type I transmembrane glycoprotein initially characterized on CD4^+^ helper T (Th)1 cells and CD8^+^ cytotoxic T cells^1^. Currently, it has been detected in several types of immune cells, including non-immune cells. As a powerful co-inhibitory molecule, Tim-3 plays a profound role in immune tolerance, autoimmune diseases^2^, ^3^, and anti-tumor and antiviral immune evasion^4^-^6^. Tim-3 acts as a brake to turn aggressive Th1 mediated auto- and alloimmune responses and mediates the depletion or dysfunction of tumor-responsive lymphocytes to promote the progression of most tumors, indicating its extensive involvement in various pathophysiological processes.

Strikingly, Tim-3 also plays an essential role in normal pregnancy, such as immune regulation, tolerance induction, and maternal-fetal cross-talk coordination^7^-^9^. Development of the semi-allogeneic fetus in the maternal uterus represents an immunological paradox in which the maternal immune system accepts the fetus expressing allogeneic paternal antigens and provides competent responses to infections. Substantial research in reproductive and transplant immunology has been conducted to address the mechanisms that prevent immune attack by a semi-allogeneic fetus with fetal histocompatibility antigens inherited from the father while protecting the mother and fetus from pathogens during pregnancy. However, the underlying mechanisms of Tim-3 in maternal-fetal tolerance have not yet been clarified. In this review, we summarize the functional regulation of Tim-3 in immunology, with a focus on its participation in maternal-fetal tolerance to provide inspiration for immunoregulation during pregnancy.

## Structure of Tim-3

**Figure 1:**
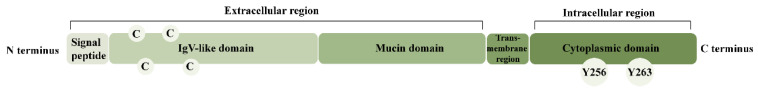
** Structure of Tim-3.** Tim-3 is a transmembrane glycoprotein comprised of 5 structural domains: a signal peptide, a characteristic IgV domain, a mucin region, a transmembrane region and an intracellular tail containing phosphorylation sites. *Abbreviations*: Tim-3, T cell immunoglobulin mucin-3; IgV, immunoglobulin variable; C, cysteine; Y, tyrosine.

Tim-3 is a transmembrane glycoprotein with conserved structure and complex cellular functions comprised of a signal peptide, a characteristic immunoglobulin (Ig) variable (V) domain, a mucin region, a transmembrane region, and an intracellular tail containing phosphorylation sites (Figure 1). Its IgV-like domain contains four conserved cysteine residues^10^; the mucin region is rich in threonine, serine, and proline, and the intracellular domain includes a tyrosine kinase phosphorylation motif^11^. Both p. Tyr82Cys Tim-3 and p. Ile97Met Tim-3 variants undergo protein misfolding and attenuate plasma membrane expression, leading to persistent immune activation^12^. Therefore, a particular amino acid sequence is indispensable for the correct localization and normal functioning of Tim-3.

## Signaling pathways of Tim-3

**Figure 2:**
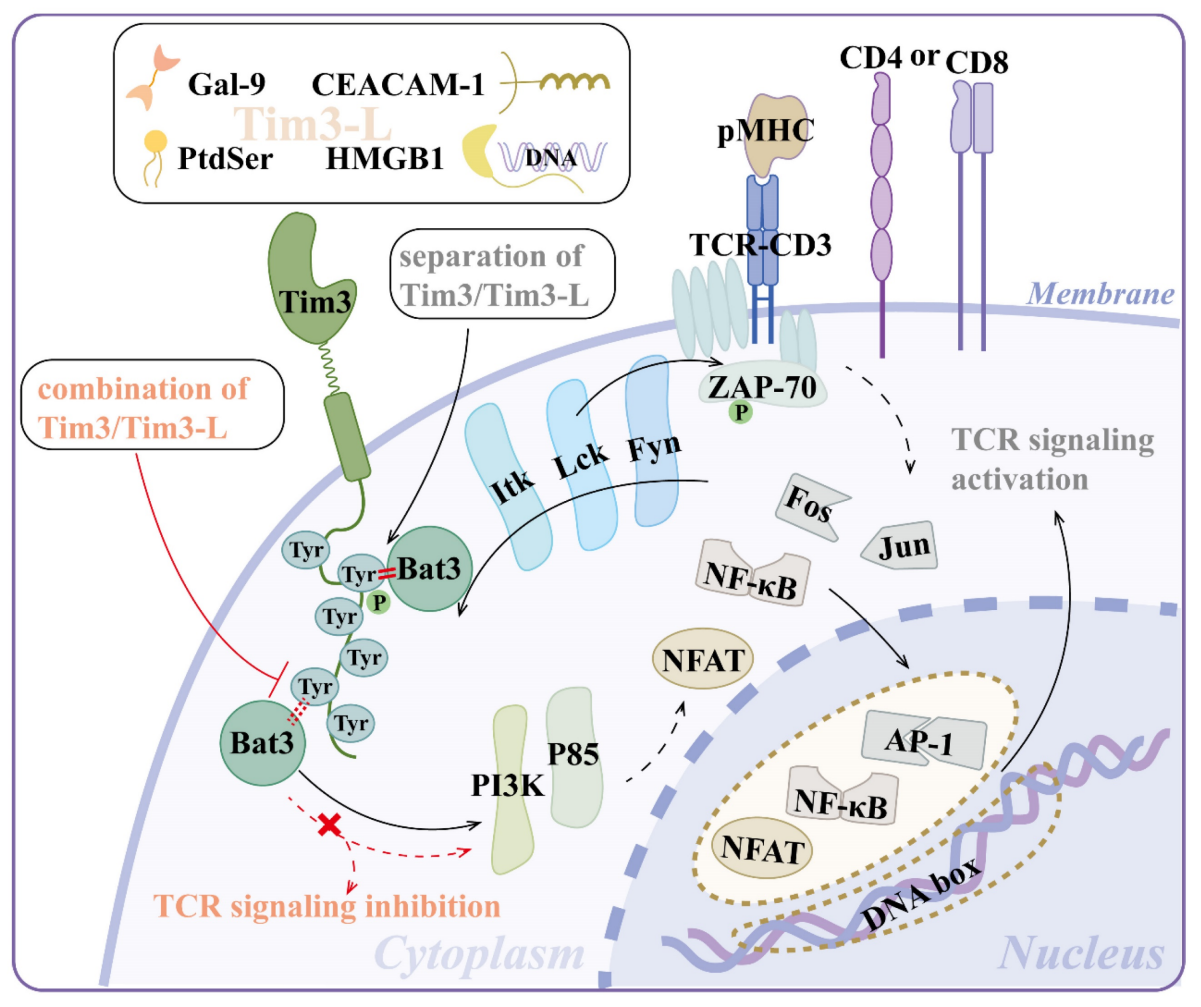
** Signal pathways of Tim-3.** (1) Separation of Tim-3 and its ligands (Gal-9, PtdSer, HMGB1, CEACAM-1) makes Bat3 bind and recruit catalytically active kinases (e.g., Lck) to the Tim-3 tail. Lck phosphorylates Tim-3. Phosphorylated Tim-3 recruits one or more SH2 domain-containing proteins (e.g., PI3K, p85 adaptor proteins), leading to increased NFAT/AP-1 and NF-κB-dependent transcription, which augments T cell activation at least under short-term stimulation. (2) The combination of Tim-3 and its ligands triggers the displacement of Bat3 and the inactivation of Lck and TCR signaling inhibition. *Abbreviations*: Tim-3, T-cell immunoglobulin mucin-3; Gal-9, galectin-9; PtdSer, phosphatidylserine; HMGB1, high-mobility group protein B1; CEACAM-1, carcinoembryonic antigen-related cell adhesion molecule-1; SH, Src homology; PI3K, phosphatidylinositol 3-kinase; NFAT, nuclear factor of activated T cell; NF-κB, nuclear factor kappa-B; TCR, T-cell receptor; pMHC, peptide-major histocompatibility complex; Tim3-L, Tim-3 ligand; Tyr, tyrosine; ZAP-70, zeta chain of T cell receptor associated protein kinase 70; AP-1, activator protein-1.

Tim-3 does not contain canonical inhibitory motifs, such as immunoreceptor tyrosine-based inhibition motifs, like programmed cell death protein-1 (PD-1) or cytotoxic T-lymphocyte antigen-4 (CTLA-4), but six well-conserved tyrosine residues on its cytoplasmic tail, implying a distinct signaling pathway (Figure 2)^13^. Tim-3 activates T cell receptor (TCR) and CD28-dependent signaling pathways that lead to increased nuclear factor of activated T cell (NFAT)/activator protein-1 (AP-1) and nuclear factor kappa-B (NF-κB)-dependent transcription, which is regulated by the phosphorylation of one or more cytoplasmic tyrosines. Mutating either Tyr256 or Tyr263 individually did not affect the activation of downstream signaling, indicating a compensatory relationship between them^14^. Paradoxically, Yin *et al.* proposed that Tim-3 deficiency resulted in activated NF-κB signaling pathway, mitochondrial damage, and cadmium nephrotoxicity exacerbation^15^.

Tim-3 can be phosphorylated by both Lck and Fyn from the Src protein tyrosine kinase family as well as Itk from the Tec family^13^. Phosphorylated Tim-3 recruits one or more Src homology (SH)2 domain-containing proteins, including phosphatidylinositol 3-kinase (PI3K) and p85 adaptor proteins, to transduce downstream signals and augment T cell activation, at least under short-term stimulation^14^. Ectopic Tim-3 expression enhanced the phosphorylation of both phospholipase C-γ1 (PLCγ1) and ribosomal protein S6, which lay downstream of the PI3K/Akt/mammalian target of the rapamycin (mTOR) pathway^16^.

Notably, among the proteins that regulate the Tim-3 signaling pathway, Bat3 (also known as Bag6 or Scythe), a ubiquitin-like chaperone protein that binds to and suppresses the function of Tim-3, serves as a functional switch for Tim-3: separation of Tim-3 and Tim-3 ligand (Tim-3L) makes Bat3 bind and recruit catalytically active Lck to the Tim-3 tail, promoting T cell signaling, activation, and expansion. The combination of Tim-3 and Tim-3L triggers the displacement of Bat3, leading to the phosphorylation of Y256 and Y263, inactivation of Lck, and TCR signaling inhibition^17^. Bat3 is involved in the mTORC2-Akt-Prdm1/B lymphocyte-induced maturation protein 1 (Blimp-1) pathway to attenuate Tim-3 induction and prevent the terminal differentiation and dysfunction of T cells^18^, ^19^. Taken together, Tim-3 and Bat3 appear to have a subtle tradeoff in immunoregulation.

## Ligands of Tim-3

Tim-3/Tim-3L interaction is essential for normal Tim-3 function. The Tim-3L concept was proposed by Sabatos *et al.* in 2003. The authors confirmed the presence of Tim-3L on CD4^+^ T cells through a secreted form of Tim-3 with only the IgV domain and demonstrated that the physiological interaction between Tim-3 and Tim-3L may limit the expansion of Th1 cell populations and induce tolerance in effector Th1 cells during normal immune responses^2^. To date, four ligands have been identified: galectin-9 (Gal-9), phosphatidylserine (PtdSer), high-mobility group protein B1 (HMGB1), and carcinoembryonic antigen-related cell adhesion molecule-1 (CEACAM-1).

### Gal-9

Galectin is a family of β-galactoside-binding lectins^20^. Gal-9 is a ubiquitously expressed tandem-repeat galectin that serves as a canonical ligand for Tim-3^21^. Tim-3/Gal-9 interaction is carbohydrate dependent^22^. Gal-9 binds to the N-terminal of the IgV domain of Tim-3 and recruits Src family kinases to the SH2 binding motif of Tim-3 to further transduce cell signaling^14^. It also facilitates the co-localization of Tim-3 and receptor phosphatases CD45/CD148 through its two carbohydrate recognition domains joined by a flexible linker, thus promoting the inhibitory function of Tim-3^23^.

### PtdSer

PtdSer is a negatively charged glycerophospholipid that is unevenly distributed in the plasma membrane and late secretory/endocytic compartments and regulates cellular activity and apoptosis^24^. As a small, nonprotein Tim-3L, PtdSer binds with a pocket on the N-terminal IgV domain of Tim-3 through an "electrostatic switching/hydrophobic anchoring" mechanism of conformational modulation^25^, ^26^, facilitating the uptake of apoptotic cells and antigen cross-presentation by dendritic cells (DCs)^27^. Delova *et al.* revealed the molecular basis and thermodynamics of Tim-3 and its interaction with PtdSer in a lipid bilayer by equilibrium and enhanced sampling molecular dynamics simulation^28^, providing cues for immunotherapeutic approaches. PtdSer induces Tim-3 phosphorylation and interferes with the PI3K/mTORC1/p-S6 signaling pathway, leading to dysregulation and inactivation of natural killer (NK) cells^29^. Consistent with this, researchers developed a photoswitchable ligand of Tim-3 that mimics the effects of PtdSer in modulating NK cell function^30^.

### HMGB1

HMGB1 is a nuclear DNA-binding protein that modulates chromosomal architecture and is a damage-associated molecular pattern^31^, ^32^. The HMGB1/Tim-3 interaction was first discovered in the defective responses of Tim-3^+^ DC to nucleic acid stimulation. HMGB1 binds to DNA released from dying cells and helps deliver this DNA to innate cells by binding to the receptors of advanced glycation end products (RAGE) and toll-like receptor (TLR)2, 4, and 9, triggering innate cell activation and pro-inflammatory cytokine production. The combination of HMGB1 and Tim-3 interferes with this process and impairs the innate immune response, thus hindering the efficacy of DNA vaccines and cytotoxic chemotherapy^33^, ^34^. During infection, HMGB1 may serve as a putative ligand for Tim-3 on CD4^+^ T cells and inhibit the NF-κB signaling pathway in Tim-3^+^CD4^+^ T cells during sepsis-induced immunosuppression^6^.

### CEACAM-1

CEACAM-1 is a single-pass type I transmembrane protein with 12 isoforms identified as novel cell surface ligands for Tim-3^35^. CEACAM-1 mediates the immunosuppressive effects of Tim-3 through binding to the CC' and FG loops of Tim-3^36^. Hajihassan *et al.* demonstrated that the IgV domain of CEACAM-1 also plays a crucial role in binding with Tim-3; the authors mutated this domain to obtain a variant with high affinity for Tim-3 to block Tim-3, which provides new strategies for immunotherapy^37^. CEACAM-1/Tim-3 cis-interaction promotes the stability of mature Tim-3 on the cell surface, which protects effector CD8^+^ T cells from premature restimulation-induced cell death (RICD) in the early stages^34^, ^38^. Recently, Chen *et al.* found that the soluble Tim-3 (sTim-3)/CEACAM-1 interaction induces terminal T cell exhaustion and attenuates CD8^+^ T cell responses to PD-1 blockade^39^. Overall, the Tim-3/Tim-3L interaction is complicated and requires further investigation.

## Tim-3 regulates immune responses

Tim-3 was present at low levels on naïve CD8^+^ T, Th1, and Th17 cells and regulatory T cells (Tregs). It is up-regulated and chiefly expressed on activated interferon (IFN)-γ-producing CD4^+^ T and CD8^+^ T cells. Tim-3 is constitutively expressed on some innate immune cells like NK cells, DCs, monocytes, macrophages (Mφs), and mast cells^40^-^45^, and even on non-immune cells like lymphoma-derived endothelial cells (ECs), human umbilical vein endothelial cells (HUVECs), and decidual stromal cells (DSCs)^46^-^48^. The wide expression profile of Tim-3 indicated its multidimensional regulation of innate and adaptive immunity and its pro- and anti-inflammatory effects.

### Tim-3 regulates innate immune responses

#### Tim-3 and Mφs

Tim-3 is constitutively expressed on unstimulated peripheral CD14^+^ monocytes but decreases rapidly upon TLR stimulation^49^. Tim-3 upregulation promotes M2 polarization of Mφ, inducing anti-tumor immune evasion in a benzene-induced acute myelocytic leukemia mouse model^50^ and development of melanoma 51, as well as infection tolerance of hepatitis virus and Listeria^52^-^54^. Recently, Wang *et al.* demonstrated that Tim-3 on Mφ promoted gut homeostasis by inhibiting neutrophil necroptosis^55^. Nonetheless, Tim-3 can promote M1 polarization in murine perihematomal brain tissue^56^. During podocyte injury and diabetic nephropathy progression, Tim-3 activates Mφ and triggers its NF-κB/tumor necrosis factor (TNF)-α signaling pathway^57^. Moreover, Tim-3 contributes to TLR-mediated NF-κB activation in IR injury-induced Mφ activation^58^. Therefore, Tim-3 exerts a dual function in Mφ regulation.

#### Tim-3 and DCs

DCs are the initiators of the adaptive immune response and the most powerful professional antigen-presenting cells (APCs) with a strong ability to activate naïve T cells. In colorectal cancer (CRC), tumor-infiltrating DCs express Tim-3, the expression of which significantly decreases with stage progression. Moreover, immature DCs express more Tim-3 than mature DCs in some CRC cases, showing close regulation of Tim-3 on tumor-infiltrating DCs^59^. Tim-3 regulates DC function partly by inhibiting inflammasome activation and stimulating interferon genes^60^, ^61^. Tim-3^+^ type 2 conventional DCs (cDCs) attenuate CD4^+^ T cell-driven anti-tumor responses, leading to poor prognosis^62^. Tim-3 blockade improves the ability of CD103^+^ cDC1 to enhance the effector function of CD8^+^ T cells in breast cancer^63^. Moreover, Tim-3 blockade potentiates interleukin (IL)-12-dependent anti-tumor immunity by facilitating spatial colocalization of CD8^+^ T cells and XCR1^+^ cDCs^64^.

#### Tim-3 and NK cells

Tim-3 is an inducible activation-limiting factor on NK cells^41^. Tim-3 represses NK cell function by inhibiting IL-12-stimualted IFN-γ production, degranulation, and cytotoxic activity^29^, ^65^ and is presented as an NK exhaustion marker in advanced melanoma, human lung adenocarcinoma, and hepatitis B virus-related hepatocellular carcinoma^19^, ^66^, ^67^. Accordingly, in severe aplastic anemia (SAA), low levels of Tim-3 contribute to the enhancement of NK cell activity, further inhibiting the immune activation state of SAA and improving the disease state^68^. In type 2 diabetes, Tim-3 overexpression in NK cells leads to impaired cytotoxicity and accelerated apoptosis of NK cells, thereby increasing the risk of cancer and infections 69. Although Tim-3 is largely regarded as an exhaustion marker on NK cells, it is noteworthy that Tim-3 could also promote NK cell activation^70^. Therefore, Tim-3 can act as an inhibitor or activator of NK cells, depending on the context and stimuli.

#### Tim-3 and mast cells

Tim-3 is constitutively expressed in mouse peritoneal mast cells and bone marrow-derived cultured mast cells and promotes IgE^+^Ag-dependent cytokine secretion of mast cells^71^. Tim-3 mediated Lyn kinase-dependent signaling pathways that modulate both immediate-phase degranulation and late-phase cytokine production downstream of FcεRI ligation in mast cell activation^72^.

### Tim-3 regulates adaptive immune responses

Tim-3 could be induced by common γ-chain cytokines (IL-2, IL-7, IL-15, and IL-21) in an antigen-independent manner on naive, effector and memory subsets of T cells in response to TCR/CD28 co-stimulation^73^. Regarding transcriptional regulation, *HAVCR2* (the protein-coding gene for Tim-3) transcription could be up-regulated by TOX, and TOX2 proteins localized apart from the nucleus in T cells^74^. Tim-3 is of great importance in tolerance induction, effector T cell (Teff) function suppression, and T cell exhaustion^75^, ^76^; however, it also enhances early T cell activation^14^. Tim-3 may be more similar to a costimulatory receptor that promotes the development of short-lived Teffs in the mTOR pathway at the expense of memory precursor development^77^. Therefore, Tim-3 is crucial for achieving optimal Teff responses. Surprisingly, Bod *et al.* also detected a unique co-inhibitory transcriptional signature, including Tim-3, on a subset of B cells in tumor-bearing mice. However, the function of Tim-3 on B cells has not yet been clearly elucidated 78.

#### Tim-3 and CD4^+^ T cells

CD4^+^ T cells express both full-length Tim-3 (flTim-3) and sTim-3 via alternative mRNA splicing. Tumor growth is positively correlated with flTim-3 levels but negatively correlated with sTim-3 levels, indicating a subtle balance between these two forms^79^. Tim-3^+^CD4^+^ T cells participate in multiple biological processes such as tumorigenesis, autoimmune diseases, and organ transplant rejection. For instance, MV3 cell-derived exosome-loaded Tim-3 inhibited the immune function of CD4^+^ T cells to accelerate melanoma progression 51. Tim-3 marked terminally differentiated and dysfunctional T cells in experimental autoimmune encephalomyelitis (EAE)^80^. Tim-3 blockade up-regulates IL-6 expression in CD4^+^ T cells and accelerates rejection^81^.

Mechanistically, Tim-3 regulates CD4^+^ T cell differentiation. Th1 and Th17 cells of patients with thyroid-associated ophthalmopathy (TAO) exhibit significantly less Tim-3 than healthy controls, suggesting a regulatory role of Tim-3 in Th1 and Th17 cells in Graves' ophthalmopathy^82^. Hepatitis C virus-infected human hepatocytes promote Tim-3 expression in co-cultured human CD4^+^ T cells and drive CD4^+^ T cells towards Tregs in a Gal-9/Tim-3 manner 83. Remarkably, Tim-3 serves as an activation marker of normal Treg function. As stronger suppressors to Teffs than Tim-3^-^ Tregs, Tim-3^+^ Tregs display increased proliferative ability with higher expression levels of IL-10, lymphocyte activation gene-3 (LAG-3), CTLA-4, and Forkhead box protein P3 (FOXP3), and inhibit IFN-γ and TNF-α secretion from peripheral blood mononuclear cells (PBMCs) more efficiently, thereby limiting pathogenic Th1/Th17 responses, and maintaining immune homeostasis more effectively^84^, ^85^. With higher expression levels of CTLA-4, PD-1, CD39, and IFN-γ receptors than Tim-3^-^ Tregs, Tim-3^+^ Tregs accumulate earlier than exhausted CD8^+^ T cells in the tumor tissue and promote tumor growth by sustaining dysfunctional CD8^+^ T cell development but inhibiting naïve T cell proliferation^4^, ^86^. Overall, Tim-3 broadly modulates CD4^+^ T cell differentiation and function by rebalancing multiple pro- and anti-inflammatory factors.

#### Tim-3 and CD8^+^ T cells

Tim-3 is found within CD8^+^ T cell lipid rafts at the immunological synapse and plays a functional role in synapse formation^23^, ^87^. Blocking Tim-3 results in significantly increased stable synapses between CD8^+^ T cells and target cells^23^. Different Tim-3Ls lead to different outcomes in CD8^+^ T cells. Gal-9 overexpression in myeloid-derived suppressor cells (MDSCs) induce an exhaustion phenotype of Tim-3^+^CD8^+^ T cells through the Tim-3/Gal-9 pathway in myelodysplastic syndrome^88^. However, another Tim-3L CEACAM-1 can promote Tim-3 surface localization and work together with Tim-3 to mitigate premature RICD in expanding effector CD8^+^ T cells during clonal expansion^38^.

Tim-3 is considered a canonical co-inhibitory receptor associated with poor tumor prognosis and is often expressed in conjunction with other co-inhibitory receptors on CD8^+^ T cells. PD-1^+^ Tim-3^+^ CD8^+^ T cells secrete less IFN-γ, TNF-α, and IL-2 compared to PD-1^+^ Tim-3^-^ or PD-1^-^Tim-3^-^ subsets. Dual blockade of Tim-3 and PD-1 during T cell priming efficiently augmented the proliferation and cytokine production of CD8^+^ T cells^89^. Nevertheless, Tim-3 is not always a risk factor for tumorigenesis. Tim-3 downregulation on CD8^+^ T cells by tumor-associated macrophage-derived IL-8 leads to impaired function and proliferation of CD8^+^ T cells. In contrast, high levels of Tim-3 contribute to stronger anti-tumor immune responses and good prognosis in CRC^90^. Therefore, Tim-3 may have a dual immunomodulatory effect on CD8^+^ T cells in different tumor microenvironments (TME).

In addition to tumorigenesis, co-expression of PD-1 and Tim-3 on CD8^+^ T cells is also associated with the upregulation of anti-atherogenic cytokines and downregulation of pro-atherogenic cytokines^91^. Blocking Tim-3 on CD8^+^ T cells mitigates anti-tuberculous immune evasion by drastically promoting IFN-γ production^92^. However, the role of Tim-3^+^CD8^+^ T cells requires further exploration.

The functional regulation of Tim-3 in non-immune cells has been explored. Lymphoma-derived ECs expressing Tim-3. Tim-3^+^ ECs modulate the T cell response to lymphoma surrogate antigens by inhibiting CD4^+^ T cell activation and Th1 polarization in an IL-6-STAT3-dependent way, thus promoting lymphoma onset, growth, and dissemination 47. Tim-3 is also expressed in HUVECs and serves as a “self-control” factor in oxidized low-density lipoprotein (ox-LDL)-triggered inflammation in HUVECs. Tim-3 protects HUVECs from ox-LDL-induced apoptosis via the JNK pathway and inhibits ox-LDL-induced inflammatory cytokine production by suppressing NF-κB activation^48^. In conclusion, Tim-3 is not restricted to immune cell markers, owing to its wide expression and intricate functions.

## Tim-3 in maternal-fetal tolerance

As the only exception to the traditional immunological process, maternal-fetal tolerance has emerged as a research hotspot in immunology. The embryo carries genetic material from both parents. The paternal human leukocyte antigen (HLA), which is carried by the fetus and expressed in the placenta, stimulates the maternal immune system and induces rejection reactions according to traditional immune theories; however, the mother immunologically recognizes these foreign fetal alloantigens and restrains the rejection response^93^. This immune regulation occurs not only in the maternal immune system but also in the local pregnancy uterus. The maternal-fetal interface is the core structure of pregnancy maintenance, with a complicated cell composition: embryonic-derived trophoblast cells, maternal-derived DSCs, and decidual immune cells (DICs) as the main components^93^. As initiators of vascular remodeling, trophoblasts recruit peripheral immune cells to the decidua and are involved in decidualization. DSCs and trophoblasts work jointly to allow local residency and functional training of DICs, thereby creating an immune-tolerant microenvironment to maintain successful pregnancy^94^, ^95^. Nevertheless, it is still largely unclear how the mother modulates the systemic and uterine immune status to tolerate the fetus and defend against pathogens simultaneously, especially the underlying molecular mechanisms. Tim-3 is an attractive candidate for exploring the pathogenesis of various cancers and has great potential in targeted immunotherapy. Interestingly, sTim-3, derived from membrane-bound Tim-3, can bind to Gal-9 to optimize immune regulation during pregnancy. Patients with unexplained recurrent pregnancy loss (RPL) express significantly higher sTim-3 but much lower Gal-9 levels than normal pregnant women with a Th1/Th2 imbalance^96^, ^97^. Given that the placenta is a pseudotumor organ^98^ and pregnancy can be likened to a semi-allograft^99^, we wondered if and how Tim-3 participates in the establishment of maternal-fetal tolerance.

### Tim-3 and DICs

DICs account for 30-40% of the total decidual cells in early pregnancy, predominantly comprised of NK cells (70%), Mφs (10-20%), T cells (10-20%), and DCs (1%). DICs display unique regulatory roles at the maternal-fetal interface, distinct from the periphery, through special activation marker expression and abundant cytokine production, facilitating tolerance bias formation 93, 95. Increasing evidence suggests that Tim-3 orchestrates innate and adaptive immune responses during pregnancy. Abnormal Tim-3 signaling or expression may cause pregnancy disorders such as RPL and preeclampsia (PE).

#### Tim-3 and decidual NK (dNK) cells

dNK cells participate in maternal-fetal tolerance because of their absolute quantitative advantage and unique phenotype (CD56^bright^CD16^dim^). dNK cells originate from peripheral recruitment and local production. Peripheral CXCR3/CXCR4^+^NK cells are recruited to the maternal-fetal interface by extra-villous trophoblast cells (EVTs) and DSCs which express corresponding chemokine ligands. In particular, human trophoblasts secrete Gal-9 and induce phenotypic alterations of peripheral NK (pNK) cells into dNK cells via the Gal-9/Tim-3 interaction^8^. In contrast, hematopoietic stem cells differentiate into NK cells in response to transforming growth factor (TGF)-β1 and IL-5 produced by DSCs and EVTs^100^-^102^.

dNK cells show higher Tim-3 expression levels than pNK cells. More than 60% of dNK cells express Tim-3 with elevated mature markers CD94, CD69, and IL-4 but lower TNF-α and perforin production in humans. Patients with RPL and abortion-prone murine models have a decreased percentage of Tim-3^+^ dNK cells^8^. Tim-3/Gal-9 signaling inhibits the degranulation of NK cells, thus attenuating NK cell cytotoxicity toward trophoblasts^103^. *Toxoplasma gondii* infection during gestation results in Tim-3 reduction on dNK cells, which unbalances dNK cell receptors, promotes cytotoxic granule production, and abnormal cytokine secretion via the PI3K-AKT and JAK-STAT signaling pathways, ultimately causing abnormal pregnancy outcomes^104^. Collectively, Tim-3 signaling is of great importance for normal dNK cell function during pregnancy.

#### Tim-3 and decidual Mφs (dMφs)

dMφs are predominantly anti-inflammatory M2 cells with a high expression of scavenger and mannose receptors and enhanced arginase activity and tissue repair capacity. dMφs are involved in the process of decidualization and spiral artery remodeling, whose dysfunction leads to adverse pregnancy outcome^105^-^107^.

Tim-3 is constitutively expressed on dMφs. Downregulation of Tim-3 on dMφs leads to disordered anti- and pro-inflammatory cytokine profiles in miscarriages^108^. Tim-3 can be downregulated by *T. gondii* infection, resulting in M1 polarization through the PI3K/AKT pathway and causing adverse pregnancy outcomes^109^. From the mechanism, an allogeneic mouse model of pregnancy showed elevated inflammatory granulocytes and Mφs, as well as pro-inflammatory cytokines at the maternal-fetal interface after Tim-3 blockade, as Tim-3 blockade impaired the phagocytic potential of dMφs, contributing to the accumulation of apoptotic bodies that elicit a local immune response^110^.

#### Tim-3 and decidual DCs (dDCs)

dDCs act as professional APCs in maternal-fetal tolerance. Tim-3 is a crucial regulator that is highly expressed on dDCs. *T. gondii* infection significantly downregulates Tim-3 on dDCs and causes dDC dysfunction, including increased CD80, CD86, MHC-II, IL-12, and TNF-α expression, but decreases indoleamine 2,3-dioxygenase (IDO) and IL-10 expression, resulting in adverse pregnancy outcomes^111^.

#### Tim-3 and decidual MDSCs

As progenitor cells of DCs, Mφs and granulocytes, MDSCs are a population of heterogeneous, immature myeloid cells with a significant inhibition ability to other immune cells^112^. During pregnancy, MDSCs accumulate in the periphery and cord blood and actively suppress the immune system^113^-^115^. MDSCs are also amplified in the decidua as a critical group of cells in maternal-fetal tolerance maintenance by promoting trophoblast implantation and angiogenesis and manipulating the function of other DICs^116^-^118^. Tim-3 blockade or depletion dramatically downregulates the functional molecules (arginase [Arg]-1 and IL-10) of decidual MDSCs and weakens their inhibitory effect on T cell proliferation through the Fyn-STAT3-CCAAT/enhancer binding protein beta (C/EBPβ) pathway, which could be reserved by Gal-9 administration^119^.

#### Tim-3 and decidual CD4^+^T (dCD4^+^T) cells

Driven by a set of transcriptional regulators and cytokines, naïve CD4^+^ T cells differentiate into distinct subsets, including Th1, Th2, Th17, and Tregs^120^. Treg expansion and Th2 bias at the maternal-fetal interface have long been considered as the main mechanisms of maternal immune tolerance toward the fetus^93^. Notably, the functional balance of dCD4^+^ T cells is closely related to immune checkpoints, including CTLA-4, PD-1, and Tim-3. In pregnant mice, decidual Tregs express higher levels of Tim-3 compared to splenic Tregs. The percentage of decidual Tim3^+^ Tregs fluctuates as gestation proceeds; it peaks at pregnancy day 6.5 (E 6.5) and accounts for approximately 60% of all decidual Tregs. Subsequently, it progressively diminished and fell to non-pregnant levels by E18.5. Tim-3 blockade decreases the proportion of Tregs in DICs, leading to embryo resorption. Consistently, in pregnant women, CD4^+^ T cells of patients with RPL at 6-9 gestational weeks expressed significantly less Tim-3 than those in normal pregnancies^121^.

Tim3^+^dCD4^+^ T cells often express other co-inhibitory receptors. Nearly 90% of decidual Tim3^+^ Tregs were PD-1 positive in pregnant mice^121^. Zhao *et al.* found that the transcription factor Blimp-1 enhances the co-expression of Tim-3 and PD-1 in decidual Tregs and boosts their immunosuppressive function in an IL-27/STAT1-dependent manner 122. Likewise, PD-1 and Tim-3 are co-expressed on dCD4^+^ T cells to shape a Th2-dominant milieu at the maternal-fetal interface during early human pregnancy. Patients with RPL display a decreased number and disordered function of Tim-3^+^PD-1^+^dCD4^+^ T cells. Blocking the PD-1 and Tim-3 pathways reduces Th2 cytokine production by dCD4^+^ T cells and increases fetal loss in mice^123^. Moreover, the Tim-3 and CTLA-4 pathways also play key roles in maintaining maternal-fetal tolerance. dCD4^+^ T cells presented higher CTLA-4 and Tim-3 expression than peripheral CD4^+^ T cells in human early pregnancy. Compared to CTLA-4^-^Tim-3^-^dCD4^+^ T cells, CTLA-4^+^Tim-3^+^dCD4^+^ T cells produced more Th2 and Treg cytokines but less pro-inflammatory cytokines (TNF-α, IFN-γ, and IL-17A). Dual blocking of CTLA-4 and Tim-3 pathways significantly abrogates Th2- and Treg-mediated fetal protection during normal pregnancy^124^. Collectively, the high temporal and spatial expression of Tim-3 on dCD4^+^ T cells during pregnancy is indispensable for Treg expansion and Th2 bias in tolerant fetuses.

#### Tim-3 and decidual CD8^+^T (dCD8^+^T) cells

dCD8^+^ T cells have been relatively poorly studied compared to dCD4^+^ T cells. CD8^+^ T cell depletion abolishes the protective effect of progesterone by altering the Th1/Th2 cytokine ratio, showing its potential role at the maternal-fetal interface^125^, ^126^. In line with dCD4^+^ T cells, there are also a cluster of Tim-3^+^ PD-1^+^dCD8^+^ T cells capable of producing more anti-inflammatory and regulatory cytokines during normal pregnancy, which is dysregulated with lower levels of anti-inflammatory cytokines (IL-4 and IL-10) but higher levels of the pro-inflammatory cytokine IFN-γ in abortion-prone mice^127^, ^128^. Additionally, Tim-3^+^CTLA-4^+^dCD8^+^ T cells showed an active status and a stronger anti-inflammatory cytokine production capacity. Blockade of the Tim-3 and CTLA-4 pathways results in dCD8^+^ T cell dysfunction and increased fetal loss^129^.

The functional regulation of Tim-3 on dCD8^+^ T cells is similar to that on dCD4^+^ T cells in some aspects: synergy with other co-inhibitory molecules (PD-1 and CTLA-4), attenuating cytotoxicity, decreasing pro-inflammatory cytokines, and increasing anti-inflammatory and regulatory cytokines, ultimately maintaining maternal-fetal tolerance. Thus, moderate expression of Tim-3 on decidual T cells and other types of DICs plays an indispensable role in maternal-fetal immune tolerance and healthy pregnancy.

### Tim-3 and DSCs

In addition to nutrient supply and endocrine function in regulating blastocyst implantation and placentation, DSCs are potential APCs that produce cytokines, present antigens, and further regulate decidual immune responses^94^, ^130^-^132^. Tim-3 was detected on DSCs. Tim-3 signaling has been identified as a self-controlling mechanism of DSCs in TLR-triggered inflammation and apoptosis during pregnancy. The DSCs of patients with RPL expressed significantly lower Tim-3 levels than those in normal pregnancies. Activation of TLR signaling up-regulated pro-inflammatory cytokines and promoted DSC apoptosis, accompanied by elevated Tim-3 expression. Tim-3, in turn, protects DSCs from TLR-mediated apoptosis in an extracellular signal-regulated kinase (ERK)1/2-dependent manner, as well as down-regulated TLR-induced inflammatory cytokine secreted by DSCs via suppressing NF-κB activation^46^. This finding highlights the role of Tim-3 in a negative feedback loop to inhibit TLR-mediated apoptosis and pro-inflammatory reactions of DSCs and provides new evidence demonstrating the key role of Tim-3 in non-immune cells in maternal-fetal tolerance.

### Tim-3 and trophoblasts

Trophoblasts also express Tim-3. Gal-9 secreted by trophoblasts interacts with Tim-3. This process, which resembles autocrine, inhibits the apoptosis and production of IFN-γ and IL-17A, promotes tube formation, invasion, and IL-4 production in trophoblasts, as well as coordinates the cross-talk between HTR8/SVneo cells and HUVECs in a JNK-dependent manner, further facilitating normal pregnancy maintenance^133^.

### Tim-3 in maternal-fetal cross-talk

**Figure 3:**
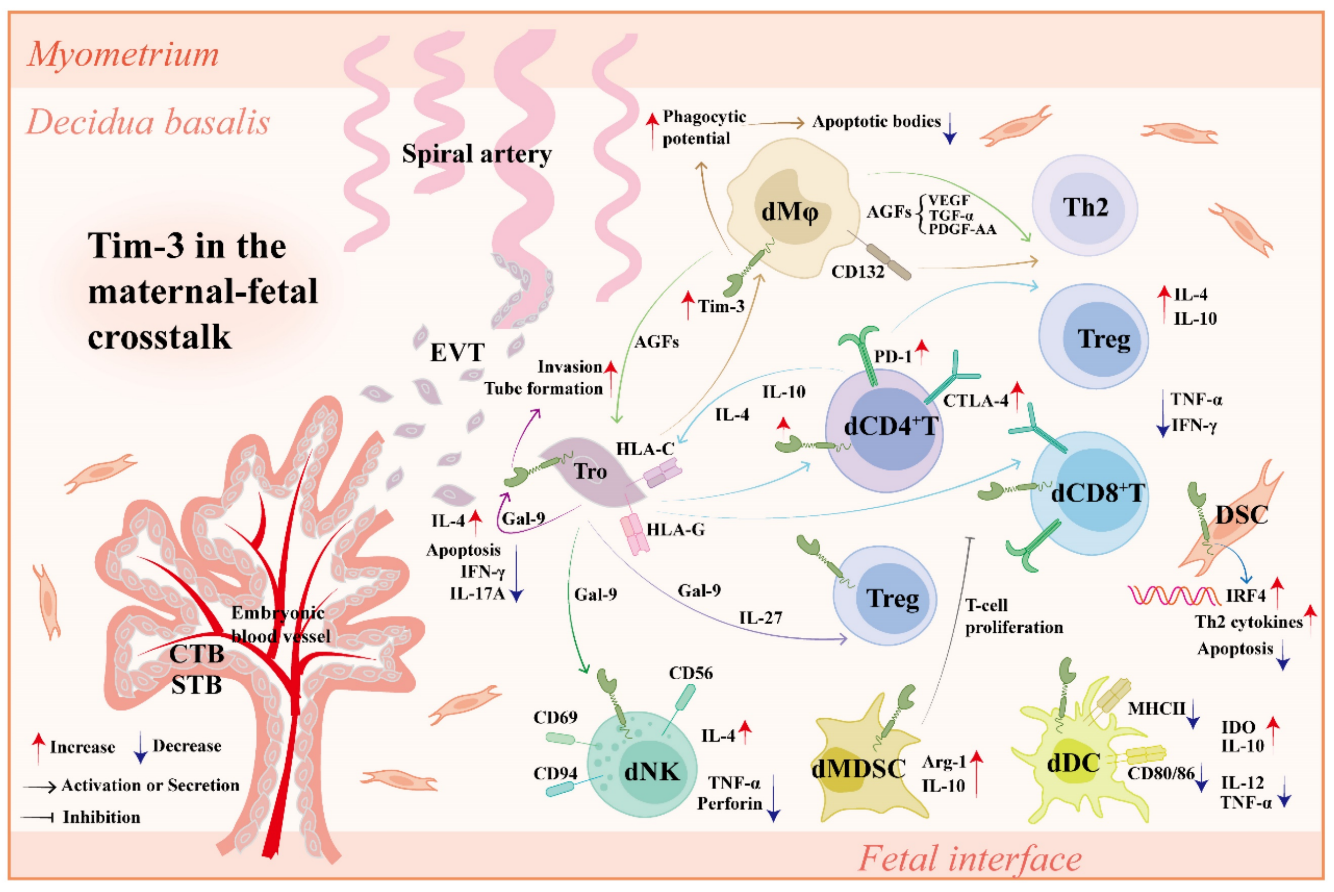
** Tim-3 in the maternal-fetal cross-talk.** Tim-3 is widely expressed on various types of cells at the maternal-fetal interface, including DICs (dNK cells, dMφs, dT cells, dDCs, dMDSCs), DSCs and Tros. In normal pregnancy, (1) Tros contribute to the higher expression of Tim-3 and other co-inhibitory molecules (e.g., CTLA-4, PD-1) on dCD4^+^T cells and dCD8^+^T cells in an HLA-C/G dependent manner. In turn, Tim-3 synergizes with other co-inhibitory molecules to improve Tro function by up-regulating lL-4 and lL-10 in dCD4^+^T cells and enhancing Tro-DIC interplay. (2) Tros produce IL-27 to up-regulate Tim-3 expression on dCD4^+^T cells, further promoting Treg differentiation and Teff/Treg balance by Tim-3/Gal-9 interaction. (3) Tros induce Tim-3 upregulation on dMφs in an HLA-C dependent manner. Tim-3^+^dMφs clear apoptotic bodies more effectively via elevated phagocytic potential to alleviate local immune response. Tim-3^+^dMφs also induce Th2 and Treg bias in dCD4^+^T cells via CD132 pathway. (4) AGFs (PDGF-AA, TGF-α, and VEGF) secreted by Tim-3^+^dMφs not only promote invasion and tube formation ability of Tros, but also promote Th2 and Treg bias in dCD4^+^T cells. (5) Tros secret Gal-9 and induce phenotype alteration of pNK cells into dNK cells via Gal-9/Tim-3 interaction. (6) Tim-3 induces functional molecules (Arg-1 and IL-10) of dMDSCs and promotes their inhibitory effect on T cell proliferation. (7) Tim-3 upregulation in dDCs results in a decrease of pro-inflammatory molecules and an increase of tolerant molecules. (8) Tim-3 increases Th2 cytokine production by DSCs in an IRF4 dependent manner, further confronting DSC apoptosis induced by LPS. (9) Tim-3 expressed on Tros interacts with Gal-9 secreted by Tros, promoting the tube formation, invasion and IL-4 production in Tros. The mechanisms above together promote maternal-fetal tolerance and pregnancy maintenance. *Abbreviations*: Tim-3, T-cell immunoglobulin mucin-3; Tros, trophoblast cells; CTLA-4, cytotoxic T-lymphocyte antigen-4; PD-1, programmed cell death protein-1; dCD4^+^T, decidual CD4^+^T; dCD8^+^T, decidual CD8^+^T; HLA, human leukocyte antigen; IL, interleukin; DIC, decidual immune cell; dMφs, decidual macrophages; Th, helper T; Treg, regulatory T cell; AGF, angiogenic growth factors; PDGF, platelet-derived growth factor; TGF-α, transforming growth factor-α; VEGF, platelet-derived growth factor; Teff, effector T cell; Gal-9, galectin-9; pNK, peripheral NK cells; dNK, decidual NK cells; Arg-1, Arginase 1; IRF4, interferon regulatory factor 4; LPS, lipopolysaccharide; CTB, cytotrophoblast; STB, syncytiotrophoblast; EVT, extra-villous trophoblast cell; TNF, tumor necrosis factor; IFN, interferon; DSC, decidual stromal cells; dMDSC, decidual myeloid-derived suppressor cell; dDC, decidual dendritic cell; MHC, major histocompatibility complex; IDO, indoleamine 2,3-dioxygenase.

Maternal-fetal cross-talk refers to a sophisticated but well-ordered interaction between trophoblasts from the fetus and DSCs/DICs from the mother. As an important immune checkpoint^134^, Tim-3 plays a crucial role in coordinating the maternal-fetal cross-talk (Figure 3).

Co-culture of peripheral CD4^+^ T cells with trophoblasts significantly increases Tim-3 expression on CD4^+^ T cells, which was abrogated by anti-HLA-C antibody administration^124^. Blocking Tim-3 pathways leads to abnormal DIC-EVT interaction, impairs placental development, and increases fetal loss, which can be rescued by IL-4 and IL-10 treatment^135^. Trophoblasts up-regulate Tim-3 expression and activate the Tim-3 signaling pathway in dCD4^+^ T cells via IL-27 and Gal-9, further contributing to Tim-3^+^ Treg accumulation and Treg/Teff balance^121^. Trophoblasts also promote Tim-3 expression on dMφs. Tim-3^+^ dMφs not only induce Th2 and Treg bias in dCD4^+^ T cells via CD132 pathway^108^ but also promote invasion and tube formation ability of trophoblasts more effectively with higher production of angiogenic growth factors (AGFs), including platelet-derived growth factor-AA (PDGF-AA), TGF-α, and vascular endothelial growth factors (VEGFs)^136^.

Recombinant Gal-9 (rGal-9) administration promotes M2 polarization and improves trophoblast function by activating Tim-3 pathway, thus alleviating adverse pregnancy outcomes in lipopolysaccharide (LPS)-induced PE-like rat model, indicating that the Tim-3/Gal-9 pathway also promotes dMφ-trophoblast dialogue to prevent the occurrence of PE^137^, ^138^. However, Hao *et al.* showed that the abnormal upregulation of the Tim-3/Gal-9 pathway could accelerate the development of PE by increasing Th1 and Th17 responses and decreasing the Th2 response^139^. Thus, the harmony and mutual benefit between trophoblasts and DICs mediated by moderate Tim-3 signaling contributes to maternal-fetal tolerance and pregnancy maintenance.

## Conclusion and prospects

In conclusion, Tim-3 serves as a crucial immune checkpoint and multifunctional immunomodulatory molecule during pregnancy by inducing maternal-fetal tolerance, enhancing maternal immunity against pathogens, and harmonizing cross-talk among different cells during maternal-fetal tolerance. Owing to non-canonical signaling, multiple ligands, and broad expression, the Tim-3 pathway is extraordinarily complicated and finely adjusted. The functional regulation of Tim-3 in reproductive immunology has been gradually unveiled, but remains to be elucidated. How does the Tim-3/Tim-3L interaction influence the immune response at the maternal-fetal interface? What endogenous or exogenous factors affect Tim-3 expression levels on cells? How can the Tim-3 signaling pathway be regulated to promote the maintenance of maternal-fetal tolerance? Further research is required to elucidate the role of Tim-3 during pregnancy.

## Abbreviations

Tim: T-cell immunoglobulin mucin
HAVCR2: hepatitis A virus cellular receptor 2
CD: cluster of differentiation
Th: helper T
IgV: immunoglobulin variable
PD-1: programmed cell death protein-1
CTLA-4: cytotoxic T-lymphocyte antigen-4
TCR: T-cell receptor
NFAT: nuclear factor of activated T cell
AP-1: activator protein-1
NF-κB: nuclear factor kappa-B
SH: Src homology
PI3K: phosphatidylinositol 3-kinase
PLC: phospholipase C
mTOR: mammalian target of Rapamycin
Tim-3L: Tim-3 ligand
Blimp-1: B lymphocyte-induced maturation protein 1
Gal-9: galectin-9
PtdSer: phosphatidylserine
HMGB1: high-mobility group protein B1
CEACAM-1: carcinoembryonic antigen-related cell adhesion molecule-1
DCs: dendritic cells
NK cells: natural killer cells
RAGE: receptor of advanced glycation end products
TLR: toll-like receptor
RICD: restimulation-induced cell death
sTim-3: soluble Tim-3
Tregs: regulatory T cells
IFN: interferon
Mφs: macrophages
ECs: endothelial cells
HUVEC: human umbilical vein endothelial cells
DSCs: decidual stromal cells
TNF: tumor necrosis factor
APC: antigen presenting cell
CRC: colorectal cancer
cDC: conventional DCs
IL: interleukin
SAA: severe aplastic anemia
Teff: effector T cell
flTim-3: full length Tim-3
EAE: experimental autoimmune encephalomyelitis
TAO: thyroid-associated ophthalmopathy
LAG-3: lymphocyte activation gene-3
FOXP3: Forkhead box protein P3
PBMC: peripheral blood mononuclear cells
MDSC: myeloid-derived suppressor cells
TME: tumor microenvironment
ox-LDL: oxidized low-density lipoprotein
HLA: human leukocyte antigen
DICs: decidual immune cells
MHC: major histocompatibility complex
RPL: recurrent pregnancy loss
PE: preeclampsia
dNK cells: decidual NK cells
EVTs: extra-villous trophoblast cells
pNK cells: peripheral NK cells
TGF: transforming growth factor
*T. gondii*: *Toxoplasma gondii*
dMφs: decidual Mφs
IDO: indoleamine 2,3-dioxygenase
Arg-1: Arginase-1
C/EBPβ: CCAAT/enhancer binding protein beta
dDCs: decidual DCs
dCD4^+^T cell: decidual CD4^+^T cell
dCD8^+^T cell: decidual CD8^+^T cell
ERK: extracellular signal regulated kinase
HTR8/SVneo cells: human-derived first trimester extravillous trophoblast cells
AGFs: angiogenic growth factors
PDGF-AA: platelet derived growth factor-AA
VEGFs: vascular endothelial growth factors
rGal-9: recombinant Gal-9
LPS: lipopolysaccharide

## References

[1] Kuchroo VK, Umetsu DT, DeKruyff RH, Freeman GJ (2003). The TIM gene family: emerging roles in immunity and disease. Nature reviews Immunology.

[2] Sabatos CA, Chakravarti S, Cha E, Schubart A, Sánchez-Fueyo A, Zheng XX (2003). Interaction of Tim-3 and Tim-3 ligand regulates T helper type 1 responses and induction of peripheral tolerance. Nature immunology.

[3] Sánchez-Fueyo A, Tian J, Picarella D, Domenig C, Zheng XX, Sabatos CA (2003). Tim-3 inhibits T helper type 1-mediated auto- and alloimmune responses and promotes immunological tolerance. Nature immunology.

[4] Sakuishi K, Ngiow SF, Sullivan JM, Teng MW, Kuchroo VK, Smyth MJ (2013). TIM3(+)FOXP3(+) regulatory T cells are tissue-specific promoters of T-cell dysfunction in cancer. Oncoimmunology.

[5] Li X, Chen Y, Liu X, Zhang J, He X, Teng G (2017). Tim3/Gal9 interactions between T cells and monocytes result in an immunosuppressive feedback loop that inhibits Th1 responses in osteosarcoma patients. International immunopharmacology.

[6] Huang S, Liu D, Sun J, Zhang H, Zhang J, Wang Q (2022). Tim-3 regulates sepsis-induced immunosuppression by inhibiting the NF-κB signaling pathway in CD4 T cells. Molecular therapy: the journal of the American Society of Gene Therapy.

[7] Zhao J, Lei Z, Liu Y, Li B, Zhang L, Fang H (2009). Human pregnancy up-regulates Tim-3 in innate immune cells for systemic immunity. Journal of immunology (Baltimore, Md: 1950).

[8] Li YH, Zhou WH, Tao Y, Wang SC, Jiang YL, Zhang D (2016). The Galectin-9/Tim-3 pathway is involved in the regulation of NK cell function at the maternal-fetal interface in early pregnancy. Cellular & molecular immunology.

[9] Zhu W, Tan YQ, Wang FY (2022). Tim-3: An inhibitory immune checkpoint is associated with maternal-fetal tolerance and recurrent spontaneous abortion. Clinical immunology (Orlando, Fla).

[10] Cao E, Zang X, Ramagopal UA, Mukhopadhaya A, Fedorov A, Fedorov E (2007). T cell immunoglobulin mucin-3 crystal structure reveals a galectin-9-independent ligand-binding surface. Immunity.

[11] Gomes de Morais AL, Cerdá S, de Miguel M (2022). New Checkpoint Inhibitors on the Road: Targeting TIM-3 in Solid Tumors. Current oncology reports.

[12] Gayden T, Sepulveda FE, Khuong-Quang DA, Pratt J, Valera ET, Garrigue A (2018). Germline HAVCR2 mutations altering TIM-3 characterize subcutaneous panniculitis-like T cell lymphomas with hemophagocytic lymphohistiocytic syndrome. Nature genetics.

[13] van de Weyer PS, Muehlfeit M, Klose C, Bonventre JV, Walz G, Kuehn EW (2006). A highly conserved tyrosine of Tim-3 is phosphorylated upon stimulation by its ligand galectin-9. Biochemical and biophysical research communications.

[14] Lee J, Su EW, Zhu C, Hainline S, Phuah J, Moroco JA (2011). Phosphotyrosine-dependent coupling of Tim-3 to T-cell receptor signaling pathways. Molecular and cellular biology.

[15] Yin G, Wang Z, Li P, Cao Y, Zhou Z, Wu W (2024). Tim-3 deficiency aggravates cadmium nephrotoxicity via regulation of NF-κB signaling and mitochondrial damage. International immunopharmacology.

[16] Ferris RL, Lu B, Kane LP (2014). Too much of a good thing? Tim-3 and TCR signaling in T cell exhaustion. Journal of immunology (Baltimore, Md: 1950).

[17] Rangachari M, Zhu C, Sakuishi K, Xiao S, Karman J, Chen A (2012). Bat3 promotes T cell responses and autoimmunity by repressing Tim-3-mediated cell death and exhaustion. Nature medicine.

[18] Tang R, Acharya N, Subramanian A, Purohit V, Tabaka M, Hou Y (2022). Tim-3 adapter protein Bat3 acts as an endogenous regulator of tolerogenic dendritic cell function. Science immunology.

[19] Yu L, Liu X, Wang X, Yan F, Wang P, Jiang Y (2021). TIGIT(+) TIM-3(+) NK cells are correlated with NK cell exhaustion and disease progression in patients with hepatitis B virus-related hepatocellular carcinoma. Oncoimmunology.

[20] Gordon-Alonso M, Bruger AM, van der Bruggen P (2018). Extracellular galectins as controllers of cytokines in hematological cancer. Blood.

[21] John S, Mishra R (2016). Galectin-9: From cell biology to complex disease dynamics. Journal of biosciences.

[22] Zhu C, Anderson AC, Schubart A, Xiong H, Imitola J, Khoury SJ (2005). The Tim-3 ligand galectin-9 negatively regulates T helper type 1 immunity. Nature immunology.

[23] Clayton KL, Haaland MS, Douglas-Vail MB, Mujib S, Chew GM, Ndhlovu LC (2014). T cell Ig and mucin domain-containing protein 3 is recruited to the immune synapse, disrupts stable synapse formation, and associates with receptor phosphatases. Journal of immunology (Baltimore, Md: 1950).

[24] Čopič A, Dieudonné T, Lenoir G (2023). Phosphatidylserine transport in cell life and death. Current opinion in cell biology.

[25] DeKruyff RH, Bu X, Ballesteros A, Santiago C, Chim YL, Lee HH (2010). T cell/transmembrane, Ig, and mucin-3 allelic variants differentially recognize phosphatidylserine and mediate phagocytosis of apoptotic cells. Journal of immunology (Baltimore, Md: 1950).

[26] Weber JK, Zhou R (2017). Phosphatidylserine-Induced Conformational Modulation of Immune Cell Exhaustion-Associated Receptor TIM3. Scientific reports.

[27] Nakayama M, Akiba H, Takeda K, Kojima Y, Hashiguchi M, Azuma M (2009). Tim-3 mediates phagocytosis of apoptotic cells and cross-presentation. Blood.

[28] Delova A, Pasc A, Monari A (2024). Interaction of the Immune System TIM-3 Protein with a Model Cellular Membrane Containing Phosphatidyl-Serine Lipids. Chemistry (Weinheim an der Bergstrasse, Germany).

[29] Tan S, Xu Y, Wang Z, Wang T, Du X, Song X (2020). Tim-3 Hampers Tumor Surveillance of Liver-Resident and Conventional NK Cells by Disrupting PI3K Signaling. Cancer research.

[30] Yang X, Li M, Qin X, Tan S, Du L, Ma C (2022). Photophosphatidylserine Guides Natural Killer Cell Photoimmunotherapy via Tim-3. Journal of the American Chemical Society.

[31] Sims GP, Rowe DC, Rietdijk ST, Herbst R, Coyle AJ (2010). HMGB1 and RAGE in inflammation and cancer. Annual review of immunology.

[32] Wang S, Zhang Y (2020). HMGB1 in inflammation and cancer. Journal of hematology & oncology.

[33] Chiba S, Baghdadi M, Akiba H, Yoshiyama H, Kinoshita I, Dosaka-Akita H (2012). Tumor-infiltrating DCs suppress nucleic acid-mediated innate immune responses through interactions between the receptor TIM-3 and the alarmin HMGB1. Nature immunology.

[34] Anderson AC, Joller N, Kuchroo VK (2016). Lag-3, Tim-3, and TIGIT: Co-inhibitory Receptors with Specialized Functions in Immune Regulation. Immunity.

[35] Huang J, Ledford KJ, Pitkin WB, Russo L, Najjar SM, Siragy HM (2013). Targeted deletion of murine CEACAM 1 activates PI3K-Akt signaling and contributes to the expression of (Pro)renin receptor via CREB family and NF-κB transcription factors. Hypertension (Dallas, Tex: 1979).

[36] Huang YH, Zhu C, Kondo Y, Anderson AC, Gandhi A, Russell A (2015). CEACAM1 regulates TIM-3-mediated tolerance and exhaustion. Nature.

[37] Hajihassan Z, Mohammadpour Saray M, Yaseri A (2023). Engineering a CEACAM1 Variant with the Increased Binding Affinity to TIM-3 Receptor. Iranian biomedical journal.

[38] Lake CM, Voss K, Bauman BM, Pohida K, Jiang T, Dveksler G (2021). TIM-3 drives temporal differences in restimulation-induced cell death sensitivity in effector CD8(+) T cells in conjunction with CEACAM1. Cell death & disease.

[39] Chen C, Zhao F, Peng J, Zhao D, Xu L, Li H (2024). Soluble Tim-3 serves as a tumor prognostic marker and therapeutic target for CD8(+) T cell exhaustion and anti-PD-1 resistance. Cell reports Medicine.

[40] Andrews LP, Yano H, Vignali DAA (2019). Inhibitory receptors and ligands beyond PD-1, PD-L1 and CTLA-4: breakthroughs or backups. Nature immunology.

[41] Ndhlovu LC, Lopez-Vergès S, Barbour JD, Jones RB, Jha AR, Long BR (2012). Tim-3 marks human natural killer cell maturation and suppresses cell-mediated cytotoxicity. Blood.

[42] Ju Y, Hou N, Meng J, Wang X, Zhang X, Zhao D (2010). T cell immunoglobulin- and mucin-domain-containing molecule-3 (Tim-3) mediates natural killer cell suppression in chronic hepatitis B. Journal of hepatology.

[43] Hastings WD, Anderson DE, Kassam N, Koguchi K, Greenfield EA, Kent SC (2009). TIM-3 is expressed on activated human CD4+ T cells and regulates Th1 and Th17 cytokines. European journal of immunology.

[44] Jones RB, Ndhlovu LC, Barbour JD, Sheth PM, Jha AR, Long BR (2008). Tim-3 expression defines a novel population of dysfunctional T cells with highly elevated frequencies in progressive HIV-1 infection. The Journal of experimental medicine.

[45] Anderson AC, Anderson DE, Bregoli L, Hastings WD, Kassam N, Lei C (2007). Promotion of tissue inflammation by the immune receptor Tim-3 expressed on innate immune cells. Science (New York, NY).

[46] Wang S, Cao C, Piao H, Li Y, Tao Y, Zhang X (2015). Tim-3 protects decidual stromal cells from toll-like receptor-mediated apoptosis and inflammatory reactions and promotes Th2 bias at the maternal-fetal interface. Scientific reports.

[47] Huang X, Bai X, Cao Y, Wu J, Huang M, Tang D (2010). Lymphoma endothelium preferentially expresses Tim-3 and facilitates the progression of lymphoma by mediating immune evasion. The Journal of experimental medicine.

[48] Qiu MK, Wang SC, Tang Y, Pan C, Wang Y, Wang SQ (2017). Tim-3 inhibits low-density lipoprotein-induced atherogenic responses in human umbilical vein endothelial cells. Oncotarget.

[49] Zhang Y, Ma CJ, Wang JM, Ji XJ, Wu XY, Moorman JP (2012). Tim-3 regulates pro- and anti-inflammatory cytokine expression in human CD14+ monocytes. Journal of leukocyte biology.

[50] Ning Q, Jian T, Cui S, Shi L, Jian X, He X (2023). Tim-3 facilitates immune escape in benzene-induced acute myeloid leukemia mouse model by promoting macrophage M2 polarization. Ecotoxicology and environmental safety.

[51] Li X, Liu Y, Yang L, Jiang Y, Qian Q (2022). TIM-3 shuttled by MV3 cells-secreted exosomes inhibits CD4(+) T cell immune function and induces macrophage M2 polarization to promote the growth and metastasis of melanoma cells. Translational oncology.

[52] Wang Z, Sun D, Chen G, Li G, Dou S, Wang R (2017). Tim-3 inhibits macrophage control of Listeria monocytogenes by inhibiting Nrf2. Scientific reports.

[53] Rong YH, Wan ZH, Song H, Li YL, Zhu B, Zang H (2014). Tim-3 expression on peripheral monocytes and CD3+CD16/CD56+natural killer-like T cells in patients with chronic hepatitis B. Tissue antigens.

[54] Gao X, Li C, Pan X, Li L, Fu J, Yao W (2013). [Expression and significance of Tim-3 on peripheral blood monocytes in patients with chronic hepatitis B]. Xi bao yu fen zi mian yi xue za zhi = Chinese journal of cellular and molecular immunology.

[55] Wang F, Zhou F, Peng J, Chen H, Xie J, Liu C (2024). Macrophage Tim-3 maintains intestinal homeostasis in DSS-induced colitis by suppressing neutrophil necroptosis. Redox biology.

[56] Yu A, Zhang X, Li M, Ye P, Duan H, Zhang T (2017). Tim-3 enhances brain inflammation by promoting M1 macrophage polarization following intracerebral hemorrhage in mice. International immunopharmacology.

[57] Yang H, Xie T, Li D, Du X, Wang T, Li C (2019). Tim-3 aggravates podocyte injury in diabetic nephropathy by promoting macrophage activation via the NF-κB/TNF-α pathway. Molecular metabolism.

[58] Guo Y, Zhang J, Lai X, Chen M, Guo Y (2018). Tim-3 exacerbates kidney ischaemia/reperfusion injury through the TLR-4/NF-κB signalling pathway and an NLR-C4 inflammasome activation. Clinical and experimental immunology.

[59] Sakuma M, Katagata M, Okayama H, Nakajima S, Saito K, Sato T (2024). TIM-3 Expression on Dendritic Cells in Colorectal Cancer. Cancers.

[60] de Mingo Pulido Á, Hänggi K, Celias DP, Gardner A, Li J, Batista-Bittencourt B (2021). The inhibitory receptor TIM-3 limits activation of the cGAS-STING pathway in intra-tumoral dendritic cells by suppressing extracellular DNA uptake. Immunity.

[61] Dixon KO, Tabaka M, Schramm MA, Xiao S, Tang R, Dionne D (2021). TIM-3 restrains anti-tumour immunity by regulating inflammasome activation. Nature.

[62] Luo J, Pang S, Hui Z, Zhao H, Xu S, Yu W (2023). Blocking Tim-3 enhances the anti-tumor immunity of STING agonist ADU-S100 by unleashing CD4(+) T cells through regulating type 2 conventional dendritic cells. Theranostics.

[63] de Mingo Pulido Á, Gardner A, Hiebler S, Soliman H, Rugo HS, Krummel MF (2018). TIM-3 Regulates CD103(+) Dendritic Cell Function and Response to Chemotherapy in Breast Cancer. Cancer cell.

[64] Gardner A, de Mingo Pulido Á, Hänggi K, Bazargan S, Onimus A, Kasprzak A (2022). TIM-3 blockade enhances IL-12-dependent anti-tumor immunity by promoting CD8(+) T cell and XCR1(+) dendritic cell spatial colocalization. Journal for immunotherapy of cancer.

[65] Wang F, Hou H, Wu S, Tang Q, Huang M, Yin B (2015). Tim-3 pathway affects NK cell impairment in patients with active tuberculosis. Cytokine.

[66] Xu L, Huang Y, Tan L, Yu W, Chen D, Lu C (2015). Increased Tim-3 expression in peripheral NK cells predicts a poorer prognosis and Tim-3 blockade improves NK cell-mediated cytotoxicity in human lung adenocarcinoma. International immunopharmacology.

[67] da Silva IP, Gallois A, Jimenez-Baranda S, Khan S, Anderson AC, Kuchroo VK (2014). Reversal of NK-cell exhaustion in advanced melanoma by Tim-3 blockade. Cancer immunology research.

[68] Ding S, Zhang T, Lei Y, Liu C, Liu Z, Fu R (2024). The role of TIM3(+) NK and TIM3(-) NK cells in the immune pathogenesis of severe aplastic anemia. Journal of translational internal medicine.

[69] Wang H, Cao K, Liu S, Xu Y, Tang L (2022). Tim-3 Expression Causes NK Cell Dysfunction in Type 2 Diabetes Patients. Frontiers in immunology.

[70] Dao TN, Utturkar S, Atallah Lanman N, Matosevic S (2020). TIM-3 Expression Is Downregulated on Human NK Cells in Response to Cancer Targets in Synergy with Activation. Cancers.

[71] Nakae S, Iikura M, Suto H, Akiba H, Umetsu DT, Dekruyff RH (2007). TIM-1 and TIM-3 enhancement of Th2 cytokine production by mast cells. Blood.

[72] Phong BL, Avery L, Sumpter TL, Gorman JV, Watkins SC, Colgan JD (2015). Tim-3 enhances FcεRI-proximal signaling to modulate mast cell activation. The Journal of experimental medicine.

[73] Mujib S, Jones RB, Lo C, Aidarus N, Clayton K, Sakhdari A (2012). Antigen-independent induction of Tim-3 expression on human T cells by the common γ-chain cytokines IL-2, IL-7, IL-15, and IL-21 is associated with proliferation and is dependent on the phosphoinositide 3-kinase pathway. Journal of immunology (Baltimore, Md: 1950).

[74] Li A, Zhang J, Zhan L, Liu X, Zeng X, Zhu Q (2024). TOX2 nuclear-cytosol translocation is linked to leukemogenesis of acute T-cell leukemia by repressing TIM3 transcription. Cell death and differentiation.

[75] Su EW, Lin JY, Kane LP (2008). TIM-1 and TIM-3 proteins in immune regulation. Cytokine.

[76] Kuchroo VK, Meyers JH, Umetsu DT, DeKruyff RH (2006). TIM family of genes in immunity and tolerance. Advances in immunology.

[77] Avery L, Filderman J, Szymczak-Workman AL, Kane LP (2018). Tim-3 co-stimulation promotes short-lived effector T cells, restricts memory precursors, and is dispensable for T cell exhaustion. Proceedings of the National Academy of Sciences of the United States of America.

[78] Bod L, Kye YC, Shi J, Torlai Triglia E, Schnell A, Fessler J (2023). B-cell-specific checkpoint molecules that regulate anti-tumour immunity. Nature.

[79] Zhu HG, Feng ZH, Geng H, Zhang GM (2005). [Expression of Tim-3 in tumor tissue and its role in the induction of tumor immune tolerance]. Xi bao yu fen zi mian yi xue za zhi = Chinese journal of cellular and molecular immunology.

[80] Zhu C, Dixon KO, Newcomer K, Gu G, Xiao S, Zaghouani S (2021). Tim-3 adaptor protein Bat3 is a molecular checkpoint of T cell terminal differentiation and exhaustion. Science advances.

[81] Boenisch O, D'Addio F, Watanabe T, Elyaman W, Magee CN, Yeung MY (2010). TIM-3: a novel regulatory molecule of alloimmune activation. Journal of immunology (Baltimore, Md: 1950).

[82] Zhao J, Lin B, Deng H, Zhi X, Li Y, Liu Y (2018). Decreased Expression of TIM-3 on Th17 Cells Associated with Ophthalmopathy in Patients with Graves' Disease. Current molecular medicine.

[83] Ji XJ, Ma CJ, Wang JM, Wu XY, Niki T, Hirashima M (2013). HCV-infected hepatocytes drive CD4+ CD25+ Foxp3+ regulatory T-cell development through the Tim-3/Gal-9 pathway. European journal of immunology.

[84] Sun H, Gao W, Pan W, Zhang Q, Wang G, Feng D (2017). Tim3(+) Foxp3 (+) Treg Cells Are Potent Inhibitors of Effector T Cells and Are Suppressed in Rheumatoid Arthritis. Inflammation.

[85] Gautron AS, Dominguez-Villar M, de Marcken M, Hafler DA (2014). Enhanced suppressor function of TIM-3+ FoxP3+ regulatory T cells. European journal of immunology.

[86] Liu Z, McMichael EL, Shayan G, Li J, Chen K, Srivastava R (2018). Novel Effector Phenotype of Tim-3(+) Regulatory T Cells Leads to Enhanced Suppressive Function in Head and Neck Cancer Patients. Clinical cancer research: an official journal of the American Association for Cancer Research.

[87] Dupré L, Aiuti A, Trifari S, Martino S, Saracco P, Bordignon C (2002). Wiskott-Aldrich syndrome protein regulates lipid raft dynamics during immunological synapse formation. Immunity.

[88] Tao J, Han D, Gao S, Zhang W, Yu H, Liu P (2020). CD8(+) T cells exhaustion induced by myeloid-derived suppressor cells in myelodysplastic syndromes patients might be through TIM3/Gal-9 pathway. Journal of cellular and molecular medicine.

[89] Lu X, Yang L, Yao D, Wu X, Li J, Liu X (2017). Tumor antigen-specific CD8(+) T cells are negatively regulated by PD-1 and Tim-3 in human gastric cancer. Cellular immunology.

[90] Zhao C, Wang D, Li Z, Zhang Z, Xu Y, Liu J (2023). IL8 derived from macrophages inhibits CD8(+) T-cell function by downregulating TIM3 expression through IL8-CXCR2 axis in patients with advanced colorectal cancer. International immunopharmacology.

[91] Qiu MK, Wang SC, Dai YX, Wang SQ, Ou JM, Quan ZW (2015). PD-1 and Tim-3 Pathways Regulate CD8+ T Cells Function in Atherosclerosis. PloS one.

[92] Wang X, Cao Z, Jiang J, Li Y, Dong M, Ostrowski M (2011). Elevated expression of Tim-3 on CD8 T cells correlates with disease severity of pulmonary tuberculosis. The Journal of infection.

[93] Arck PC, Hecher K (2013). Fetomaternal immune cross-talk and its consequences for maternal and offspring's health. Nature medicine.

[94] Zhou WH, Du MR, Dong L, Yu J, Li DJ (2008). Chemokine CXCL12 promotes the cross-talk between trophoblasts and decidual stromal cells in human first-trimester pregnancy. Human reproduction (Oxford, England).

[95] Moffett A, Loke C (2006). Immunology of placentation in eutherian mammals. Nature reviews Immunology.

[96] Wu M, Zhu Y, Zhao J, Ai H, Gong Q, Zhang J (2015). Soluble costimulatory molecule sTim3 regulates the differentiation of Th1 and Th2 in patients with unexplained recurrent spontaneous abortion. International journal of clinical and experimental medicine.

[97] Grossman TB, Minis E, Loeb-Zeitlin SE, Bongiovanni AM, Witkin SS (2021). Soluble T cell immunoglobulin mucin domain 3 (sTim-3) in maternal sera: a potential contributor to immune regulation during pregnancy. The journal of maternal-fetal & neonatal medicine: the official journal of the European Association of Perinatal Medicine, the Federation of Asia and Oceania Perinatal Societies, the International Society of Perinatal Obstet.

[98] Murdock TA, Varghese A, Xing D, Schoolmeester JK, Alexander C, Baergen RN (2022). Bizarre Chorionic-type Trophoblast in Second-trimester and Third-trimester Placentas: Clinicopathologic Characterization of a Placental Pseudoneoplastic Lesion. The American journal of surgical pathology.

[99] Trowsdale J, Betz AG (2006). Mother's little helpers: mechanisms of maternal-fetal tolerance. Nature immunology.

[100] Vacca P, Vitale C, Montaldo E, Conte R, Cantoni C, Fulcheri E (2011). CD34+ hematopoietic precursors are present in human decidua and differentiate into natural killer cells upon interaction with stromal cells. Proceedings of the National Academy of Sciences of the United States of America.

[101] Keskin DB, Allan DS, Rybalov B, Andzelm MM, Stern JN, Kopcow HD (2007). TGFbeta promotes conversion of CD16+ peripheral blood NK cells into CD16- NK cells with similarities to decidual NK cells. Proceedings of the National Academy of Sciences of the United States of America.

[102] Du MR, Wang SC, Li DJ (2014). The integrative roles of chemokines at the maternal-fetal interface in early pregnancy. Cellular & molecular immunology.

[103] Sun J, Yang M, Ban Y, Gao W, Song B, Wang Y (2016). Tim-3 Is Up-regulated in NK Cells during Early Pregnancy and Inhibits NK Cytotoxicity toward Trophoblast in Galectin-9 Dependent Pathway. PloS one.

[104] Li T, Cui L, Xu X, Zhang H, Jiang Y, Ren L (2021). The Role of Tim-3 on dNK Cells Dysfunction During Abnormal Pregnancy With Toxoplasma gondii Infection. Frontiers in cellular and infection microbiology.

[105] Wang WJ, Hao CF, Lin QD (2011). Dysregulation of macrophage activation by decidual regulatory T cells in unexplained recurrent miscarriage patients. Journal of reproductive immunology.

[106] Sica A, Mantovani A (2012). Macrophage plasticity and polarization: in vivo veritas. The Journal of clinical investigation.

[107] Douglas NC, Zimmermann RC, Tan QK, Sullivan-Pyke CS, Sauer MV, Kitajewski JK (2014). VEGFR-1 blockade disrupts peri-implantation decidual angiogenesis and macrophage recruitment. Vascular cell.

[108] Li M, Sun F, Xu Y, Chen L, Chen C, Cui L (2022). Tim-3(+) decidual Mφs induced Th2 and Treg bias in decidual CD4(+)T cells and promoted pregnancy maintenance via CD132. Cell death & disease.

[109] Zhang D, Ren L, Zhao M, Yang C, Liu X, Zhang H (2019). Role of Tim-3 in Decidual Macrophage Functional Polarization During Abnormal Pregnancy With Toxoplasma gondii Infection. Frontiers in immunology.

[110] Chabtini L, Mfarrej B, Mounayar M, Zhu B, Batal I, Dakle PJ (2013). TIM-3 regulates innate immune cells to induce fetomaternal tolerance. Journal of immunology (Baltimore, Md: 1950).

[111] Xie H, Li Z, Zheng G, Yang C, Liu X, Xu X (2022). Tim-3 downregulation by Toxoplasma gondii infection contributes to decidual dendritic cell dysfunction. Parasites & vectors.

[112] Veglia F, Perego M, Gabrilovich D (2018). Myeloid-derived suppressor cells coming of age. Nature immunology.

[113] Köstlin N, Hofstädter K, Ostermeir AL, Spring B, Leiber A, Haen S (2016). Granulocytic Myeloid-Derived Suppressor Cells Accumulate in Human Placenta and Polarize toward a Th2 Phenotype. Journal of immunology (Baltimore, Md: 1950).

[114] Zhao H, Kalish F, Schulz S, Yang Y, Wong RJ, Stevenson DK (2015). Unique roles of infiltrating myeloid cells in the murine uterus during early to midpregnancy. Journal of immunology (Baltimore, Md: 1950).

[115] Rieber N, Gille C, Köstlin N, Schäfer I, Spring B, Ost M (2013). Neutrophilic myeloid-derived suppressor cells in cord blood modulate innate and adaptive immune responses. Clinical and experimental immunology.

[116] Pang B, Hu C, Li H, Nie X, Wang K, Zhou C (2023). Myeloidderived suppressor cells: Escorts at the maternal-fetal interface. Frontiers in immunology.

[117] Li C, Chen C, Kang X, Zhang X, Sun S, Guo F (2020). Decidua-derived granulocyte macrophage colony-stimulating factor induces polymorphonuclear myeloid-derived suppressor cells from circulating CD15+ neutrophils. Human reproduction (Oxford, England).

[118] Li C, Zhang X, Kang X, Chen C, Guo F, Wang Q (2020). Up-regulated TRAIL and Reduced DcR2 Mediate Apoptosis of Decidual PMN-MDSC in Unexplained Recurrent Pregnancy Loss. Frontiers in immunology.

[119] Qi H, Li Y, Liu X, Jiang Y, Li Z, Xu X (2023). Tim-3 regulates the immunosuppressive function of decidual MDSCs via the Fyn-STAT3-C/EBPβ pathway during Toxoplasma gondii infection. PLoS pathogens.

[120] Zeng W, Liu Z, Liu X, Zhang S, Khanniche A, Zheng Y (2017). Distinct Transcriptional and Alternative Splicing Signatures of Decidual CD4(+) T Cells in Early Human Pregnancy. Frontiers in immunology.

[121] Hu X, Zhu Q, Wang Y, Wang L, Li Z, Mor G (2020). Newly characterized decidual Tim-3+ Treg cells are abundant during early pregnancy and driven by IL-27 coordinately with Gal-9 from trophoblasts. Human reproduction (Oxford, England).

[122] Zhao SJ, Hu XH, Lin XX, Zhang YJ, Wang J, Wang H (2024). IL-27/Blimp-1 axis regulates the differentiation and function of Tim-3+ Tregs during early pregnancy. JCI insight.

[123] Wang S, Zhu X, Xu Y, Zhang D, Li Y, Tao Y (2016). Programmed cell death-1 (PD-1) and T-cell immunoglobulin mucin-3 (Tim-3) regulate CD4+ T cells to induce Type 2 helper T cell (Th2) bias at the maternal-fetal interface. Human reproduction (Oxford, England).

[124] Wang S, Chen C, Li M, Qian J, Sun F, Li Y (2019). Blockade of CTLA-4 and Tim-3 pathways induces fetal loss with altered cytokine profiles by decidual CD4(+)T cells. Cell death & disease.

[125] Blois SM, Joachim R, Kandil J, Margni R, Tometten M, Klapp BF (2004). Depletion of CD8+ cells abolishes the pregnancy protective effect of progesterone substitution with dydrogesterone in mice by altering the Th1/Th2 cytokine profile. Journal of immunology (Baltimore, Md: 1950).

[126] Shao L, Jacobs AR, Johnson VV, Mayer L (2005). Activation of CD8+ regulatory T cells by human placental trophoblasts. Journal of immunology (Baltimore, Md: 1950).

[127] Xu YY, Wang SC, Lin YK, Li DJ, Du MR (2017). Tim-3 and PD-1 regulate CD8(+) T cell function to maintain early pregnancy in mice. The Journal of reproduction and development.

[128] Wang SC, Li YH, Piao HL, Hong XW, Zhang D, Xu YY (2015). PD-1 and Tim-3 pathways are associated with regulatory CD8+ T-cell function in decidua and maintenance of normal pregnancy. Cell death & disease.

[129] Wang S, Sun F, Li M, Qian J, Chen C, Wang M (2019). The appropriate frequency and function of decidual Tim-3(+)CTLA-4(+)CD8(+) T cells are important in maintaining normal pregnancy. Cell death & disease.

[130] Shao Q, Liu X, Huang Y, Chen X, Wang H (2020). Human Decidual Stromal Cells in Early Pregnancy Induce Functional Re-Programming of Monocyte-Derived Dendritic Cells via Cross-talk Between G-CSF and IL-1β. Frontiers in immunology.

[131] Du L, Deng W, Zeng S, Xu P, Huang L, Liang Y (2021). Single-cell transcriptome analysis reveals defective decidua stromal niche attributes to recurrent spontaneous abortion. Cell proliferation.

[132] Li X, Shi J, Zhao W, Huang X, Cui L, Liu L (2022). WNT16 from decidual stromal cells regulates HTR8/SVneo trophoblastic cell function via AKT/beta-catenin pathway. Reproduction (Cambridge, England).

[133] Li M, Peng X, Qian J, Sun F, Chen C, Wang S (2021). Galectin-9 regulates HTR8/SVneo function via JNK signaling. Reproduction (Cambridge, England).

[134] Starling S (2017). Immune tolerance: A mother's greatest gift is TIM3. Nature reviews Immunology.

[135] Li M, Sun F, Qian J, Chen L, Li D, Wang S (2021). Tim-3/CTLA-4 pathways regulate decidual immune cells-extravillous trophoblasts interaction by IL-4 and IL-10. FASEB journal: official publication of the Federation of American Societies for Experimental Biology.

[136] Cui L, Sun F, Xu Y, Li M, Chen L, Chen C (2023). Tim-3 Coordinates Macrophage-Trophoblast Cross-talk via Angiogenic Growth Factors to Promote Pregnancy Maintenance. International journal of molecular sciences.

[137] Li ZH, Wang LL, Liu H, Muyayalo KP, Huang XB, Mor G (2018). Galectin-9 Alleviates LPS-Induced Preeclampsia-Like Impairment in Rats via Switching Decidual Macrophage Polarization to M2 Subtype. Frontiers in immunology.

[138] Hu XH, Li ZH, Muyayalo KP, Wang LL, Liu CY, Mor G (2022). A newly intervention strategy in preeclampsia: Targeting PD-1/Tim-3 signaling pathways to modulate the polarization of decidual macrophages. FASEB journal: official publication of the Federation of American Societies for Experimental Biology.

[139] Hao H, He M, Li J, Zhou Y, Dang J, Li F (2015). Upregulation of the Tim-3/Gal-9 pathway and correlation with the development of preeclampsia. European journal of obstetrics, gynecology, and reproductive biology.

